# Preoperative Assessment and Perioperative Management of Resectable Gallbladder Cancer in the Era of Precision Medicine and Novel Technologies: State of the Art and Future Perspectives

**DOI:** 10.3390/diagnostics12071630

**Published:** 2022-07-05

**Authors:** Gianluca Cassese, Ho-Seong Han, Yoo-Seok Yoon, Jun Suh Lee, Jai Young Cho, Hae-Won Lee, Boram Lee, Roberto Ivan Troisi

**Affiliations:** 1 Department of Surgery, Seoul National University College of Medicine, Seoul National University Bundang Hospital, Seongnam 13620, Korea; gianluca.cassese91@gmail.com (G.C.); yoonys@snubh.org (Y.-S.Y.); rudestock@gmail.com (J.S.L.); jycho@snubh.org (J.Y.C.); lansh@hanmail.net (H.-W.L.); boramlee0827@snubh.org (B.L.); roberto.troisi@unina.it (R.I.T.); 2 Department of Clinical Medicine and Surgery, Division of Minimally Invasive and Robotic HPB Surgery, Federico II University Hospital, 80131 Naples, Italy

**Keywords:** gallbladder cancer, preoperative assessment, endoscopic ultrasounds, fine needle aspiration biopsy, laparoscopic surgery, ctDNA, NGS

## Abstract

Gallbladder carcinoma (GBC) is a rare malignancy, with an estimated 5-year survival rate of less than 5% in the case of advanced disease. Surgery is the only radical treatment for early stages, but its application and effectiveness depend on the depth of tumoral invasion. The extent of resection is usually determined according to the T-stage. Therefore, an early and correct preoperative assessment is important for the prognosis, as well as for the selection of the most appropriate surgical procedure, to avoid unnecessary morbid surgeries and to reach the best outcomes. Several modalities can be used to investigate the depth of invasion, from ultrasounds to CT scans and MRI, but an ideal method still does not exist. Thus, different protocols are proposed according to different recommendations and institutions. In this scenario, the indications for laparoscopic and robotic surgery are still debated, as well as the role of new technologies such as next-generation sequencing and liquid biopsies. The aim of this article is to summarize the state of the art current modalities and future perspectives for assessing the depth of invasion in GBC and to clarify their role in perioperative management accordingly.

## 1. Introduction

Gallbladder cancer (GBC) is a rare but highly aggressive malignancy, with a higher incidence in some countries in Asia, Latin America, and Eastern Europe [1]. It is often diagnosed at an advanced stage, because of both the lack of clinical symptoms in the early stages and the anatomical peculiarity of the absence of a submucosal layer [2]. However, the widespread use of ultrasonography (US) and computed tomography (CT) has increased the detection of early-stage GB cancer [3]. 

Surgery is the only effective treatment, but it only has satisfying results for the early stage of the disease, while medical therapy has shown very low effectiveness [4]. According to the largest series available in the literature, patients with the T1 stage can reach a 5-year survival rate of 85.9%, as compared to 19.2% and 14.1% of patients with T3 and T4 stages, respectively [5]. However, the biggest difference in survival rates is shown between T1 and T2 gallbladder cancer (beyond the subserosal layer), where overall survival (OS) drops to 56% [5]. Thus, the extent of surgical resection, as well as the need for medical therapy, is determined by the T-stage. The likelihood of microscopic liver metastases or nodal involvement, which have been reported as important prognostic factors, are linked to the T stage as well. Finally, in the surgical treatment of T2 gallbladder cancer, tumor biology may play a greater role in outcome than does the extent of resection [6]. Therefore, accurate prognostic stratification is important to determine both the curability and the appropriate extent of surgery.

Surgery is indicated only for the early-stage disease, which is typically asymptomatic. The possible scenarios associated with early GBC can be identified as a casual finding after abdominal imaging, usually performed for other reasons, or as an incidental malignancy at pathologic examination after cholecystectomy [7]. While incidentally identified carcinoma accounts for less than 1% of all cholecystectomy specimens, the occurrence of incidental findings at imaging is rising thanks to the increasing use of imaging tests for screening other pathologies [8]. Unfortunately, the majority of cases of gallbladder carcinoma (75%) are still diagnosed when the disease is no longer resectable [8]. 

Several modalities can be used to investigate the depth of invasion of resectable GBCs, from ultrasounds to CT scans and MRI, but an ideal method still does not exist. Thus, different protocols are proposed according to different recommendations and institutions. 

The aim of this review is to summarize the state of the art current modalities for investigating the depth of invasion in GBC and to clarify their actual and future role within clinical protocols and clinical management.

## 2. Preoperative Staging 

### 2.1. Ultrasonography

The transabdominal US is the first imaging modality for evaluating gallbladder disease. The US has a sensitivity of 85% and overall accuracy of 80% in diagnosing gallbladder cancer, but only in locally advanced disease [9]. In cases where the tumor is flat or in the setting of cholelithiasis, the US may miss the lesion [10]. In addition, the US is not accurate in presenting the full extent of the disease, since it is notably limited in the assessment of lymph node involvement, as well as of metastases to abdominal organs and peritoneum [11]. 

Endoscopic ultrasonography (EUS) is considered to be superior to US and CT in terms of GB imaging thanks to its capacity to highlight the different layers of the GB wall and provide high-resolution images [12,13]. The EUS images were classified into four categories by Fujita et al. [14]: type A is a pedunculated mass with an intact neighboring wall; type B is a large-based mass with an irregular surface and intact outer hyperechoic layer; type C has an irregular outer hyperechoic layer; and type D has the outer hyperechoic layer disrupted by a mass echo. Each of the four categories of EUS was shown to be well correlated with the histologic depth of invasion, with type C corresponding to T2 and type D to T3–4 [15]. However, in type B, there can be some understaging of T2 tumors [15].

The main limitations for EUS are still represented by its operator dependence and invasiveness. Furthermore, they provide surgeons with unfamiliar anatomic images.

Recently, the accuracy in the diagnosis of invasion of the subserosa level (ss) was improved to as high as 93.8% by Sugimoto et al., thanks to a specific EUS sign, namely the disrupted lateral hyperechoic layer (LHEL) [16]. In this study conducted on 49 patients, the diagnosis of ss invasion based on an irregular LHEL (narrowed or thickened) had the highest sensitivity and accuracy among the different EUS imaging parameters tested (sensitivity 97.1%, specificity 86.7%, and accuracy 93.8%). Furthermore, a very interesting aspect was that by using this parameter, the diagnostic accuracy of GBC with ss invasion was not significantly different between EUS specialists and beginners. Even if the routine use of EUS within many international guidelines is still not recommended [17], we strongly suggest performing it, and also taking into account the last expert consensus about GBC surgical treatment [18].

### 2.2. Computed Tomography

Several studies have investigated the CT criteria and their diagnostic accuracy for the T-stage of GBC [3,19]. However, there are still discordant results in diagnostic performance and inter-observer variability.

A very recent study, including 159 patients, showed an overall accuracy of 87% for a preoperative assessment of the depth of invasion by using a multidetector computed tomography scan (MDCT) by experienced radiologists. However, the inter-observer agreement was not very high (*κ* = 0.36), with a sensitivity for T-stage determination varying from 37% to 73%, and specificity from 75% to 91%, mostly depending on the radiologist’s expertise. The accuracy of MDCT for differentiating T1 and T2 GB cancer is also limited and there is considerable interobserver variability. Accordingly, several authors suggest combining different methods. Interestingly, some authors proposed to combine imaging and biochemical data to create a more accurate and less operator-dependent preoperative system, showing how tumor diameter and preoperative CEA values were independent predictors of a T stage ≥ to pT2, and building a regression formula with an area under the receiver operating characteristics (ROC) curve of the obtained pT2 predictive score as high as 0.873 [20]. 

Nevertheless, MDCT plays a fundamental role in the preoperative assessment of lymph node involvement, peritoneal implants, and vascular invasion, being considered the most accurate modality to determine resectability [21]. 

Finally, in the case of suspected occult peritoneal or omental and/or LN metastases, positron emission tomography-computed tomography (PET-CT) has shown to be a valuable preoperative tool to rule out a metastatic disease [22,23].

### 2.3. Magnetic Resonance Imaging

MRI should be systematically performed in case of GBC, thanks to a higher sensitivity than CT for detecting direct hepatic invasion, varying in literature from 87.5% to 100% [24]. In fact, dynamic contrast-enhanced fat-suppressed T1-weighted gradient-echo MRI improves the characterization of the gallbladder wall, allowing a better evaluation of the liver parenchyma for tumor infiltration [9]. A careful assessment of the gallbladder wall must look for the smooth profile of the wall itself throughout its entire internal and external course; a loss of tissue planes with adjacent structures can be an early sign of direct invasive disease. An improved assessment of focal liver invasion was reported by using gadoxetic acid-enhanced MRI for the staging of gallbladder carcinoma by Hwang et al. [25]. In our experience, conventional gadolinium-based contrast agents are typically sufficient for the preoperative staging, with T2-weighted fat-saturated or T2-weighted effective in detecting invasion of adjacent liver parenchyma. A recent study on 86 patients published by Kim et al. showed an overall accuracy of MRI for the final T-staging of gallbladder cancer of 84.9% by an experienced radiologist, with 10.5% of overstaging, and a consistent inter-observer variability. Understaging was less frequent (4%). Regarding the ability to differentiate T1 from T2 and T2 from T3, the sensitivities and the specificities varied in the range of 78.95 and 94.03%. 

Finally, another indication for MRI is the detection of biliary obstruction, for which sensitivity reaches 69% and is higher than the CT-scan, providing a more accurate study of biliary tree anatomy and eventual invasion [26]. In fact, MR cholangiopancreatography is useful in the detection of bile duct invasion and evaluation of biliary obstruction, allowing avoidance of more invasive exams such as invasive endoscopic retrograde cholangiopancreatography (ERCP) [24,26,27]. 

### 2.4. EUS-Guided Fine Needle Aspiration 

Although EUS-guided Fine Needle Aspiration (EUS-FNA) is highly useful and widely used for pancreatic carcinoma and other gastrointestinal lesions, several concerns exist about its routinary use for GBC, due to the high risk of tumoral dissemination and biliary fistula [28]. Endoscopic bile duct biopsy can be an option if a preoperative pathological diagnosis of GBC is necessary when a biliary stricture is present [29]. However, when a biliary stricture is absent, it is often necessary to rely on the cytological examination of bile, which is less accurate [30]. 

Regarding the evaluation of lymph node involvement, FNA has been reported to reach a high diagnostic performance of EUS-FNA in gallbladder lesions, with a sensitivity, specificity, and diagnostic accuracy of 80–100%, 100%, and 83–100%, respectively [31]. Furthermore, when compared with Endoscopic Retrograde Cholangiopancreatography-guided (ERC) FNA, EUS-FNA had a sensitivity as high as 96%, showing no complications [32]. However, the role of EUS-FNA is not clarified by the latest international guidelines and it is currently not recommended [33].

## 3. Clinical Management According to the Depth of Invasion

The depth of invasion (T-stage) and lymph node involvement (N-stage) are the main prognostic factors in patients with GBC [34]. T1 carcinomas confined to the lamina propria (pT1a) and the muscle layer (pT1b) have a risk of lymph node metastasis <10%, with a 5-year survival rate of 82–100% after cholecystectomy [35]. Furthermore, the risk of tumor invasion and the modality of cancer spread has been thought to be influenced by tumor position, due to the particular anatomical relationships of the gallbladder with half of its body lying on the liver and the other half protruding into the abdominal cavity. Nevertheless, the clinical significance of the exact location of the lesion has not yet been clarified, and the current edition of the American Joint Committee on Cancer staging system for gallbladder cancer does not take it into account [36]. 

According to the latest guidelines, as far as the optimal extent of resection is concerned, simple cholecystectomy is sufficient for the treatment of T1a GBC because of the extremely low risk of both lymphatic and distant metastases [33]. 

**Figure 1:**
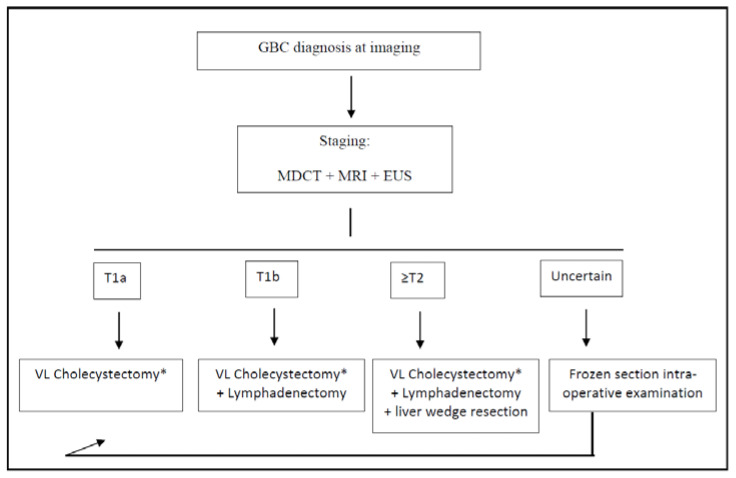
Treatment algorithm for the post-imaging diagnosis of GBC. * Frozen section intra-operative examination of cystic duct margin is always performed during cholecystectomy; if positive, common bile duct resection is performed. GBC: gallbladder cancer; VL: video-laparoscopic; MDCT: multi-detector computed tomography; MRI: magnetic resonance imaging; and EUS: endoscopic ultrasounds.

For T1b GBC, it is still a matter of debate if whether simple cholecystectomy or extended surgery is adequate for treatment [37]. Kim et al. recently published an international multicenter retrospective study involving 272 patients with incidental T1b GBC, showing no differences in the overall 5-year disease-specific survival between simple cholecystectomy and extended resection [38]. Among two meta-analyses, one concluded that the extended resection had no benefit, while the other concluded that an extended surgery with hepatic resection prolonged survival [39,40]. Considering that the aforementioned study involved only incidentally diagnosed GBC, according to our experience and to the latest NCCN Guidelines, in our institution, a cholecystectomy plus lymphadenectomy of stations 9, 12, and 13 is formally planned for all T1b staged GBC [33] (Figure 1). This is the recommended extent of lymphadenectomy according to several experts, as stated in the consensus conference held in Seoul in 2017 [41]. Furthermore, we strongly suggest removing the gallbladder with its serosa and cystic plate, en bloc with the aforementioned lymph nodes, taking care not to squeeze or break the latter ones during the manipulations.

**Figure 2:**
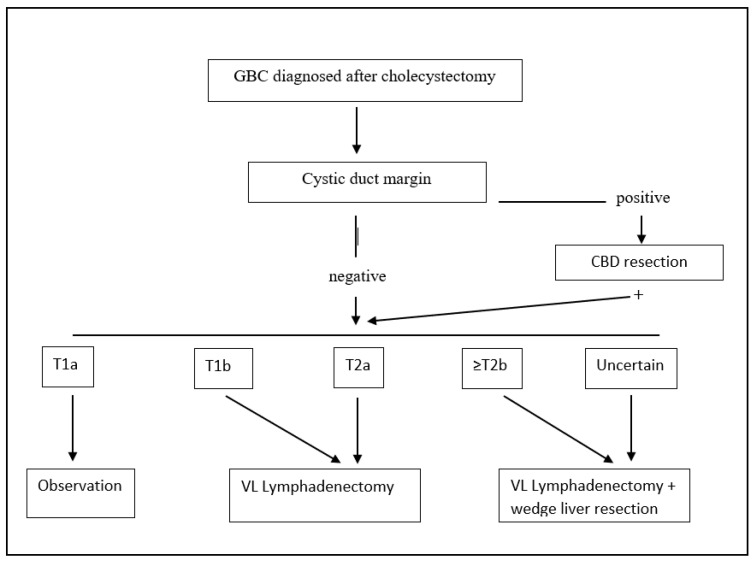
Treatment algorithm for incidental post-cholecystectomy diagnosis of GBC. GBC: gallbladder cancer; VL: video-laparoscopic; and CBD: common bile duct.

When dealing with pT2 or more advanced tumors, many authors have advocated extended surgery with hepatic resection and that lymphadenectomy is indispensable due to high rates of liver and lymph node metastases, even if some authors have reported that 40.5% of patients with inapparent pT2 tumors survive more than 5 years after cholecystectomy alone [42], and other centers suggest performing an extended cholecystectomy only if the tumor is located on the liver side [43]. The most recent and largest multicenter study on 937 patients showed that extended cholecystectomy was marginally superior to simple cholecystectomy, including liver resection and adequate node dissection [44]. Furthermore, tumor location was not shown to be an independent prognostic factor. The common bile duct (CBD) should be resected only in case the cystic duct margin of resection comes positive [45]. Similar improved outcomes are reached in patients with pT2 GBC incidentally found after cholecystectomy, when compared to cholecystectomy alone [46] (treatment algorithm for incidentally found GBC is shown in Figure 2). Nonetheless, there is still a considerable variation in the literature about the definition of “radical cholecystectomy” which usually includes a cholecystectomy, a wedge resection of the gallbladder fossa with a rim of at least 2 cm non-neoplastic liver tissue, and regional lymph node dissection in an en bloc fashion, but for other authors, should also include a CBD resection.

Finally, an interesting recent article showed acceptable results from extended resection for more advanced diseases (where extended resection was defined as a major hepatectomy, a pancreatoduodenectomy, or both) [47]. The median OS after extended surgery for advanced GBC was 12.8 months, but postoperative morbidity was significant (>60%) [47]. A 2-year survival was achieved for 30% of patients, and a 5-year survival period for 15%. Factors associated with reduced survival were the involvement of common bile duct, liver, perineural and perivascular tissue, and preoperative jaundice. The presence of preoperative obstructive jaundice was confirmed to be associated with reduced OS and increased postoperative morbidity as well by a wide meta-analysis by Dasari B.D.M et al. which concluded that surgery for such cases can be performed, but only after careful evaluation and counseling [48].

### 3.1. Clinical Management of Incidentally Diagnosed GBC

The majority of patients in low-incidence countries are incidentally diagnosed after cholecystectomy for a presumed benign disease. Despite the differences in incidence and disease presentation, disease-specific survival has been reported to be independent of geography [49]. Incidental GBCs tend to be associated with more favorable pathologic characteristics such as a lower T-stage, compared to non-incidentally diagnosed GBCs [50]. Results from a retrospective analysis on the risk factors for incidental GBC include cholelithiasis, obesity, multiparity, and Salmonella or Helycobacter infection [51]. 

The accurate pathologic staging of incidental GBC within the cholecystectomy specimen is fundamental for correct management. A correct risk stratification is the key, and it is related to patient survival and the probability of residual cancer in the cholecystectomy bed or portal lymph nodes [52,53]. The primary predictor of residual disease is once again the pathologic T-stage of the tumor, where patients with T2 or T3 tumors can have between a 10–57% and 36–77% incidence of residual disease, respectively [54,55]. However, appropriate pathologic evaluation of the T-stage can often be complicated by the initial non-oncologic resection of the gallbladder specimen. Thus, a score to predict the presence of residual disease based on the T-stage and other pathologic features was developed by Ethun et al. and was found to be correlated with OS [56].

We recommend re-resection of the tumor bed with partial hepatectomy only for select patients with GBC a T2b or higher stage (Figure 2). A complete staging with lymphadenectomy of stations number 9, 12, and 13 should be performed in all patients staged ≥ T1b. 

The timing of re-resection after the discovery of incidental GBC is still debated. A previous study showed that only the TNM stage, not the time interval between cholecystectomy and re-resection, is a prognostic factor for patients [57]. On the other hand, some authors reported an optimal interval time of 4 to 8 weeks, as re-resection before or after this interval was associated with worse outcomes, even when accounting for tumor stage [58].

The role of chemotherapy in the perioperative setting for patients with GBC has always been a matter of debate, with several randomized trials and a meta-analysis of twenty studies including 6712 patients concluding in favor of the administration of adjuvant therapy after resection, even if without a univocal consensus on the ideal regimen [59]. 

Until recently, the adjuvant regimen was extrapolated thanks to the evidence from the ABC-02 trial, which involved 149 patients with GBC, and showed that a combination of cisplatin-gemcitabine had an OS of 11.7 months versus 8.1 months with gemcitabine alone [60]. The recent PRODIGE 12 study tried to investigate the value of gemcitabine-oxaliplatin compared to placebo in the adjuvant setting, but it was reported as a negative trial [61]. The BILCAP (BILiary CAPecitabine) randomized controlled trial in 447 patients with resected biliary tract malignancies reported that 6 months of adjuvant capecitabine improved overall survival compared to placebo in the intent-to-treat analysis [62], even if the trial reported a statistically non-significant *p*-value (*p* = 0.097). Thus, this regimen has now become the standard of care recommendation after the re-resection of incidental GBC [63]. Currently, the ACTICAA-1 study is assessing the value of chemotherapy augmentation with gemcitabine-cisplatin compared to capecitabine [64]. 

The utility of adjuvant radiation has not been proven, and currently, chemoradiation is recommended only in the case of microscopically disease-positive surgical margins (R1 resections) [63]. Furthermore, chemoradiation in the setting of GBC was studied recently in 2015 with the Southwest Oncology Group (SWOG) S0809 Phase II trial which reported a well-tolerated adjuvant regimen of gemcitabine and capecitabine with radiotherapy and showed a 2-year OS of 67% and a median OS of 35 months [65]. Efficacy of this regimen would need to be further tested in a Phase III setting, ideally with pre-planned subgroup analysis for incidentally diagnosed GBC. 

There currently is no evidence for the use of chemotherapy in the neoadjuvant setting prior to re-resection. 

Several other limitations still exist, especially considering that all previous trials tested all biliary tract malignancies together, despite each tumor type having distinct molecular signatures and biologic behaviors. Accordingly, we are waiting for the results of the OPT-IN phase III trial which is comparing neoadjuvant gemcitabine-cisplatin + adjuvant capecitabine to adjuvant capecitabine alone for patients with T2-T3 incidental GBC [66].

### 3.2. Role of Minimally Invasive Surgery

The development of new technologies in the last three decades has resulted in tremendous advances in the surgical treatment of GBC as well. In particular, laparoscopic surgery has widely spread as a safe treatment approach for many GI tract cancers [67]. Surprisingly, consensus guidelines on GBC treatment are based mostly on data reported before 2000. Previous studies reported poor outcomes for laparoscopic surgery on GBC, including port site metastasis and poorer survival [68,69]. However, more recent studies, involving patients with early GBC, have reported significantly improved outcomes after laparoscopic treatment when compared with open surgery [70].

In 2004, we first started a prospective comparative study investigating the outcomes of laparoscopic treatment of resectable GBC [71]. After 10 years, we published our encouraging long-term outcomes on 83 patients with a median follow-up of 60 months, with an overall survival (OS) rate of 90.7%, and a disease-specific 5-year survival rate of 94.2% [72]. There were no cases with local recurrence at the trocar’s sites, at the lymphadenectomy site, or the gallbladder bed. Since then, we concluded that laparoscopy for GBC is an oncologically safe option and we systematically used the minimally invasive approach. A further study involving 247 patients with GBC comparing open and laparoscopic surgery, showed no statistical difference between the OS in the two groups, as well as in the subgroup analysis according to T2 stage and lymph node involvement (T2N0 vs. T2N1) [73]. There was no significant difference between the open group and the laparoscopic group, in both the T2N0 subgroup and T2N1 subgroup. 

In 2019, the first expert consensus meeting on laparoscopic surgery for GBC treatment was held in Seoul, Korea, and the first consensus statement was established, after performing an international survey among the most expert surgeons in the field of GBC surgery [18,41]. The consensus meeting concluded that laparoscopic surgery does not worsen the prognosis of patients with resectable GBC and that the postoperative and survival outcomes of highly selected patients were favorable, even when dealing with liver and bile duct resection. The majority of surgeons agreed that laparoscopic surgery has acceptable safety and effectiveness for suspicious or early GBC, and that laparoscopic extended cholecystectomy has comparable outcomes compared to open surgery, in selected patients with GBC, in expert centers. Lymph-node dissection can be performed to the same extent in laparoscopic surgery as in the open procedure in selected GBC patients, with few intraoperative and postoperative complications. Similarly, laparoscopic surgery for lymphadenectomy and liver resection can also be performed in case of re-operation after incidental post-cholecystectomy GBC findings [74]. Even if there are more papers about the outcomes from T3 or higher stages, the quality of the evidence is still low for these stages, and an objective definition of the expert centers is still needed [75].

### 3.3. Role of Robotic Surgery

The advent of robotic surgery is certainly another milestone in the evolution of minimally invasive surgery, even if the benefits are still a matter of debate for many subspecialties. With regard to GBC, several reports have been published [76]. Goel et al. showed their results on 27 patients undergoing robotic surgery for GBC, with lower intraoperative bleedings and length of hospital stay when compared with an open approach, confirming its safety and feasibility [77]. However, some issues need to be further investigated. Firstly, data about the oncological outcomes are still lacking, mainly due to a short follow-up in the few available studies. Secondly, data about T3 or higher stages of GBC are still scarce. Regarding the possible advantages, some authors suggest how the robotic approach could be useful thanks to the high degree of freedom that can facilitate the reconstruction phase in the case of bile duct resection [78]. The economic concerns are a matter of debate, especially when considering that surgery for T1 GBC consists of cholecystectomy with/without a lymphadenectomy, for which the expenses linked to the robotic platform are still too high. Furthermore, when liver resection is required, parenchymal transection may be difficult, as the cavitron ultrasonic surgical aspirator is not available in robotic surgery. 

To date, there is insufficient evidence of the benefit of the robotic system over laparoscopic surgery, in terms of extended cholecystectomy.

### 3.4. Indocyanine Green Fluorescence

Near-infrared fluorescence (NIRF) imaging using indocyanine green (ICG) is a new real-time navigation tool widely used in laparoscopic and open hepatobiliary surgery [79]. In 2018, Seo et al. first published their experience with ICG-guided fluorescence, during four cases of open radical cholecystectomy, by injecting the dye inside the cystic artery through a conventional open approach. Similarly, Yu et al. published a case of ICG-guided laparoscopic surgery for a T3 staged GBC using 0.1 mg/kg ICG for tumor and biliary tree visualization during the operative procedure [80]. The tumor and biliary tree were clearly visualized by utilizing the green fluorescence dye. The patient was successfully operated on through the laparoscopic approach, undergoing radical cholecystectomy with liver wedge resection and lymphadenectomy. According to this case, ICG has been proposed to be helpful in the achievement of a negative margin and lymphatic clearance around the biliary tree. Finally, ICG-guided surgery for GBC was also reported for the robotic approach, where authors proposed its usefulness for obtaining negative cystic duct margin and lymphatic clearance around the biliary tree, especially in complex re-explorative biliary surgery [81].

Further studies are needed, but thanks to its safety and its high visive impact, ICG-guided surgery seems to have only benefits for the surgical treatment of GBC.

## 4. Novel Approaches for GBC Staging and Management

### 4.1. Circulating Free DNA 

As previously stated, GBC diagnosis is often too tardive, thus, there is a need to find a method for early diagnosis, as well as more accurate methods to preoperatively stage the depth of invasion in order to correctly plan eventual surgery and avoid an over-or under-treatment [82]. Currently, there is no single specific tumor biomarker for the diagnosis and prognosis of GBC. Tumor biomarkers such as cancer antigens (CA-125, 19.9), carcinoembryonic antigen (CEA), and alpha-phetoprotein (AFP) have been extensively used for other hepatobiliary malignancies, even if they have low specificity [83]. In this scenario, the estimation of serum levels of the cf-DNA levels in GBC can probably play an interesting role, as already demonstrated for several other neoplasms [84,85,86,87]. 

Kumari et al. first showed the role of liquid biopsy for GBC, showing how circulating levels of long DNA fragments deriving from tumor necrosis reached a sensitivity, specificity, and diagnostic accuracy of 80.0%, 86.1%, and 82.2% respectively [88]. On the contrary, global DNA-methylation showed a low accuracy. Furthermore, circulating free DNA (Cf-DNA) integrity had an AUC of 0.813 at a cut-off of >0.3422 to discriminate the stage I-II vs. III-IV patients with sensitivity, specificity, and diagnostic accuracy of 90.20%, 77.78%, and 85.0% respectively [88]. For the discrimination of LN involvement, the sensitivity, specificity, and diagnostic accuracy reached up to 83.87%, 55.0%, and 72.5%, respectively, using a cut-off value of >0.4049 [88]. Thus, it seems that this method provides good sensitivity, specificity, and diagnostic accuracy for differentiating GBC from other diseases, in particular when using a chemiluminescence DNA biosensor method, possibly leading to the use of this approach in the coming years to obtain an early diagnosis [89].

Large prospective studies in different stages of GBC are still needed to establish the use of cfDNA in everyday clinical practice.

### 4.2. Next-Generation Sequencing

Next-generation sequencing (NGS) is becoming more and more appealing as a research and clinical tool to enable drug selection based on genome biomarkers. The genetic analyses of EUS-FNA specimens from the hepatobiliary tract using targeted NGS have already been reported [90,91]. Furthermore, driver genes have been identified in gallbladder and biliary tract cancers, such as the ERBB2, PIK3CA, BRCA1/2, and FGFR2 [92,93,94]. Thus, specimens obtained by EUS-FNAB can be used for next-generation sequencing of GBC, possibly becoming useful in the diagnosis, staging, and treatment selection for gallbladder cancers.

No targeted therapy is currently available for GBC, but mutations in ATM, ERBB2, and PIK3CA, and amplifications in ERBB2, are currently targetable with FDA-approved drugs in other solid tumors [95]. Therefore, these alterations are potential targets in GBC and might be included in future molecular testing panels for tailored treatments. Furthermore, another gene is correlated with clinical response to immunotherapy in several tumors, namely the TMB-H, including biliary tract neoplasms, as well as detected checkpoint inhibitors, such as pembrolizumab, can be treatment options [96,97]. Another gene linked with the response to immune checkpoint inhibitors is MSI, for which pembrolizumab is already available in the USA, showing an objective response rate of 41% in patients treated for cholangiocarcinoma [98]. Therefore, even if they are mutated in a small proportion of GBC, both TMB-H and MSI-H are interesting biomarkers for tailored treatment.

Nine clinical trials are currently evaluating targeted therapies directed at frequently occurring genetic alterations in GBC, but not one has concluded, underlining how this field has a relative delay compared to other tumors [96]. As a matter of fact, the organization of clinical trials including patients with GBC is logistically challenging since GBC is very rare in most Western countries. Furthermore, there is a large inter-tumor heterogeneity of GBC. A clinical trial that tests agents in different cancer types with the same genetic alteration, namely the so-called basket trials, could provide a solution to this challenge in future studies.

## 5. Conclusions

GBC is a malignancy with an extremely poor prognosis in case of a late diagnosis. For resectable disease, proper preoperative staging can be critical to avoid unnecessary morbidity. The combination of CT, MRI, and EUS can provide an adequate range of information for a correct staging, overcoming the inter-observer variability, thanks to the technological advances obtained with these methods.

This review tries to clarify for the first time all the evidence about the whole perioperative management of the possible different scenarios of GBC, according not only to the most recent literature but also to the authors’ wide experience. Accordingly, we tried to define the role and the extent of lymphadenectomy, as well as radical cholecystectomy. The minimally invasive surgical approach has been stated by experts as a viable option in centers with high volume and expertise, being able to guarantee the advantages linked to a lower length of stay and perioperative bleeding, without affecting oncological outcomes for low stage cases.

For the more advanced disease, there is a strong need for advances in medical therapy, where innovative techniques such as cf-DNA and NGS assays are likely to play an important role in the coming years.

Further studies with an appropriate design are still needed to solve several open issues, in particular about the role of surgery in frail and elderly patients, as well as the role of perioperative chemotherapy.

## Data Availability

Not applicable.
